# Supplementary material for: A Novel Nutritional Index Serves as A Useful Prognostic Indicator in Cardiac Critical Patients Requiring Mechanical Circulatory Support
Source: Nutrients. 2019 Jun 24;11(6):1420. doi: 10.3390/nu11061420 (PMC6627834; doi:10.3390/nu11061420)
Supplement: Supplementary file 1 [file nutrients-11-01420-s001.pdf]

Supplementary Table 1: Unadjusted univariate Cox proportional hazard analyses for all-cause (a) and cardiovascular (b) mortality

a All-cause mortality

	HR	95.0% CI		P
**lower sBP <102 mmHg (median)**	**2.66**	**1.71**	**4.13**	**<0.0001**
**1SD higher sBP**	**0.56**	**0.44**	**0.70**	**<0.0001**
Higher heart rate >86 bpm (median)	1.06	0.73	1.55	0.755
**Concomittant use of VA-ECMO**	**5.55**	**3.80**	**8.12**	**<0.0001**
Diabetes	0.91	0.60	1.40	0.68
**Stage 3-5 CKD**	**3.29**	**2.12**	**5.10**	**<0.0001**
**1SD higher eGFR**	**0.59**	**0.48**	**0.72**	**<0.0001**
**Acute coronary syndrome**	**0.51**	**0.35**	**0.74**	**<0.0001**
**Cardiogenic shock**	**2.14**	**1.18**	**3.91**	**0.013**
Congestive heart failure	1.24	0.66	2.30	0.507
**GNRI-T3**	**0.35**	**0.20**	**0.60**	**<0.0001**
**TCBI-T3**	**0.23**	**0.12**	**0.45**	**<0.0001**
**1SD higher GNRI**	**0.58**	**0.49**	**0.69**	**0<0.0001**
**1SD higher TCBI**	**0.29**	**0.15**	**0.59**	**0.001**

b Cardiovascular mortality

	HR	95.0% CI		P
**lower sBP <102 mmHg (median)**	**3.32**	**2.05**	**5.37**	**<0.0001**
**1SD higher sBP***	**0.51**	**0.40**	**0.64**	**<0.0001**

Higher heart rate >86 bpm (median)	1.04	0.70	1.55	0.843
**Concomittant use of VA-ECMO**	**5.90**	**3.98**	**8.75**	**<0.0001**
Diabetes	1.01	0.66	1.56	0.954
**Stage 3-5 CKD**	**3.44**	**2.16**	**5.47**	**<0.0001**
**1SD higher eGFR**	**0.59**	**0.48**	**0.73**	**<0.0001**
**ACS**	**0.53**	**0.36**	**0.78**	**0.001**
**Cardiogenic shock**	**2.09**	**1.12**	**3.91**	**0.021**
Congestive heart failure	0.96	0.47	1.98	0.92
**GNRI-T3**	**0.38**	**0.22**	**0.65**	**<0.0001**
**TCBI-T3**	**0.23**	**0.11**	**0.45**	**<0.0001**
**1SD higher GNRI**	**0.59**	**0.50**	**0.71**	**<0.0001**
**1SD higher TCBI**	**0.29**	**0.14**	**0.60**	**0.001**

Supplementary Table 2: Adjusted multivariate Cox proportional hazard analysis for all-cause mortality of age, TCBI and markers of organ function

	HR	95% CI	p-value
Age, 1 year older	1.00	0.97-1.03	0.86
**TCBI, 1SD higher**	**0.29**	**0.10-0.81**	**0.02**
BNP, 100 pg/ml higher	0.96	0.86-1.07	0.44
**eGFR, 10 ml/min/1.73m^2 higher**	**0.82**	**0.73-0.93**	**0.001**
**Total bilirubin, 1 mg/dl higher**	**1.81**	**1.11-2.95**	**0.02**

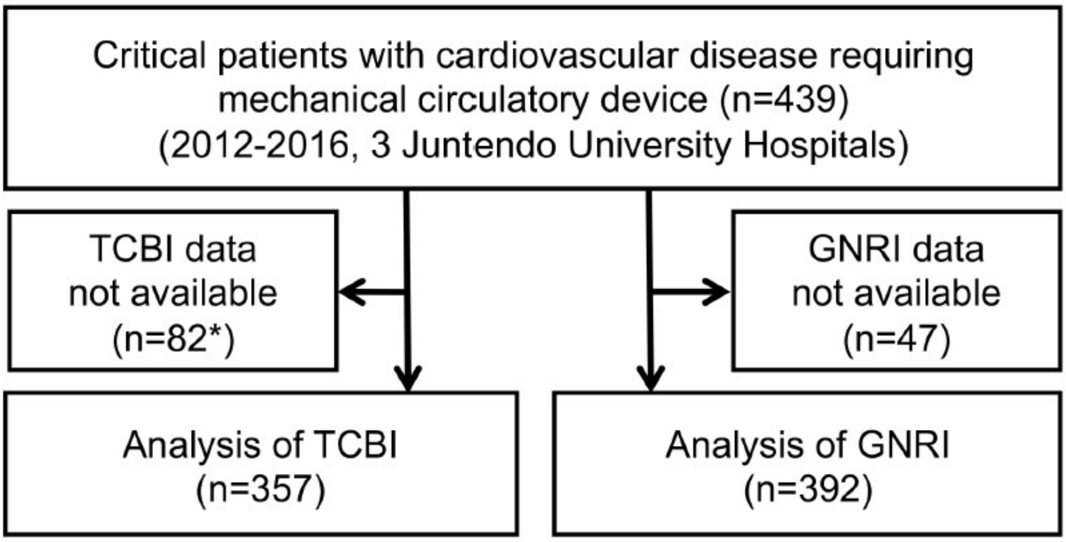

Supplementary Figure 1: Levels of nutritional indices in patients who required MCS devices and with stable CAD

GNRI and TCBI in patients who required MCS devices and with stable CAD. Patients in the MCS group were further divided into two groups, ACS (-) and (+). **: p<0.001, ***: p<0.0001.

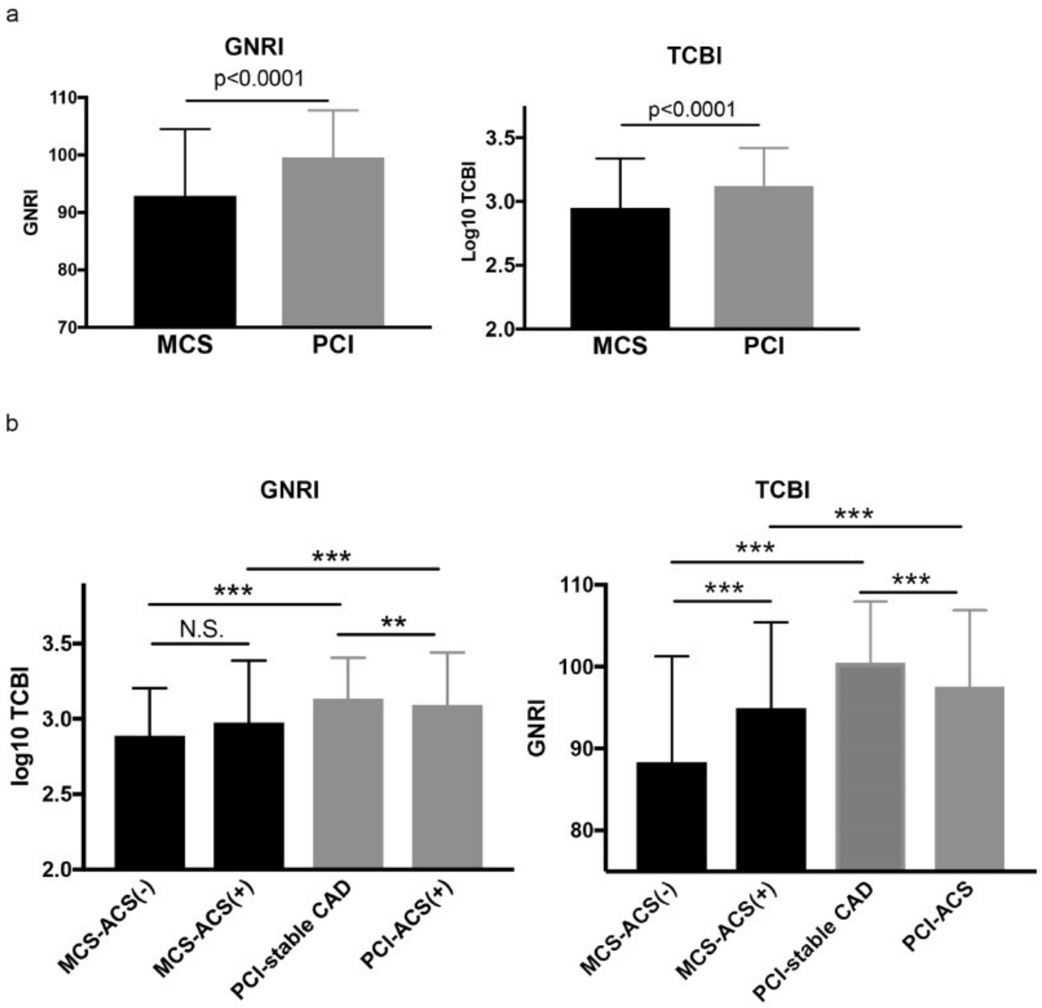

Supplementary Figure 2: Higher GNRI was associated with reduced risk of all-cause and cardiovascular mortalities

Kaplan-Meier analysis and log-rank comparisons to assess cumulative incidence rates of both all-cause (left panel) and cardiovascular (right panel) mortality in accordance with tertile of GNRI. *<0.05, **<0.01, ***<0.001 in log-rank comparison vs. TCBI-T1 and ¶<0.05, ¶¶<0.01, ¶¶¶<0.001 vs. TCBI-2.

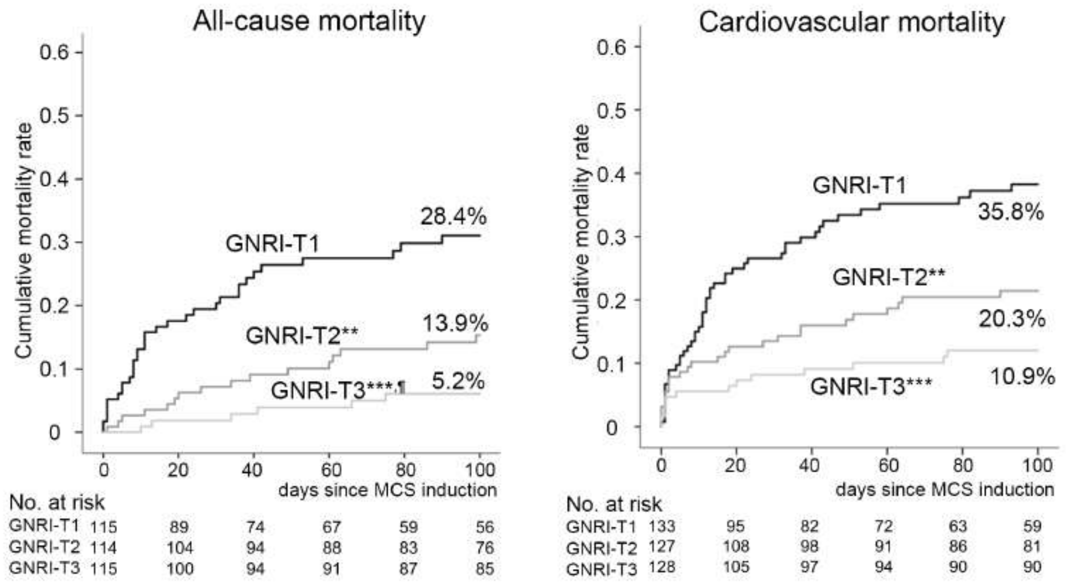

Supplementary Figure 3: Adjusted hazard ratios for cardiovascular mortality of high TCBI and GNRI

Forest plots of higher GNRI and TCBI as a nominal variable (highest tertile, T3, upper panel) and continuous variable (1 standard deviation (SD) higher, lower panel) for cardiovascular mortality. HR: hazard ratio, CI: confidence interval, P: p-value.
